# Innovative approaches for augmenting dielectric properties in cross-linked polyethylene (XLPE): A review

**DOI:** 10.1016/j.heliyon.2024.e34737

**Published:** 2024-07-22

**Authors:** A. Nazrin, T.M. Kuan, Diaa-Eldin A. Mansour, Rizwan A. Farade, A. Mohd Ariffin, M.S. Abd Rahman, Noor Izzri Bin Abdul Wahab

**Affiliations:** a Department of Science and Technology, Faculty of Humanities, Management and Science, Universiti Putra Malaysia Bintulu Campus, Bintulu, 97008, Sarawak, Malaysia; b Institute of Power Engineering, Department of Electrical and Electronics Engineering, College of Engineering, Universiti Tenaga Nasional, Jalan IKRAM-UNITEN, 43000, Kajang, Selangor, Malaysia; c Department of Electrical Power and Machines Engineering, Faculty of Engineering, Tanta University, Tanta, 31511, Egypt; d Department of Electrical Power Engineering, Egypt-Japan University of Science and Technology (E-JUST), New Borg El-Arab City, Alexandria, 21934, Egypt; e Advanced Lightning, Power and Energy Research (ALPER), Department of Electrical and Electronics Engineering, Faculty of Engineering, University Putra Malaysia, 43400, Serdang, Malaysia

**Keywords:** Power cables, Cross-linked polyethylene, Nanocomposites, Voltage stabilisers, Antioxidant

## Abstract

Throughout the history of power systems, power cables have been used to securely and efficiently distribute electrical energy to the destined locations. Cross-linked polyethylene (XLPE), a commonly used insulator in high-voltage cables, have several desirable properties, such as low dielectric loss, high dielectric constant, high thermal conductivity, enhanced thermal stability, and superior resistance against electrical stress. However, further improvements of XLPE's performance are needed. The incorporation of large specific surface area nanoparticles, such as boron nitride nanosheets and graphene oxide, exhibited a great potential in enhancing XLPE's properties. These nanoparticles create numerous trapping sites, even at small loading levels, due to their large interfacial regions. In addition, voltage stabilisers with polar groups can scavenge high-energy electrons generated by local electric fields, thereby inhibiting the electrical tree growth. Another important aspect of enhancing XLPE's dielectric performance is the inclusion of antioxidants with phenolic groups. These antioxidants react with peroxyl radicals, mitigating their harmful effects. This review summarises the effects of nanoparticles, voltage stabilisers, antioxidants, and polymer amalgamation on dielectric performance of XLPE as an insulation material. The major challenges in dielectric insulation such as breakdown voltage strength, electrical tree growth, structural defect, space charge accumulation, and thermal aging are addressed.

## 1 Introduction

The transmission of electricity to neighbourhoods, industries, and commercial centres requires an efficient, secure, and reliable system. Transmission lines are responsible for carrying electrical energy in large quantities through electric conductors to distribution substations. Power cables are considered an efficient and safe methodology to transmit electrical energy. These cables can stretch for hundreds to thousands of kilometres from the energy generation site to reach the intended consumers. Given the labour-intensive work and high costs involved, such systems are designed to have a long lifespan and require minimal maintenance. The insulation of transmission cables plays a crucial role in ensuring the functionality of the entire system by preventing electrical leakages, ensuring safe and effective power transfer [1]. Insulation can be described as a material possessing the ability to resist the flow of electrical current. Nowadays, polymer-based materials had been extensively used as an insulating material due their high dielectric strength, low dielectric loss and satisfactory mechanical strength.

The earliest known record on cables or wires was reported in 1812, when Pavel Schilling, a Russian diplomat, adopted rubber-varnished-insulated wires to remotely detonate mines. The use of copper insulated with porcelain or glass emerged in the 1850s and 1860s. In 1881, Thomas A. Edison developed the first power distribution system using copper rods wrapped in jute and inserted into iron pipes filled with a wax compound. It was not until 1890, where Dr Ferranti successfully implemented high voltage insulated conductor in the first truly modern power station for London Electric Supply Corporation [2]. Fluid/oil-impregnated paper cables goes back in 1872, but the fully established design emerged in the early 20th century. However, due to their drawbacks, such as high dielectric loss, environmental pollution, and high operating costs, they were considered inferior as insulating materials. Subsequently, organic-based insulators like natural rubber and natural resins were used, but their limitations in terms of processability and voltage levels led to the development of synthetic materials. Synthetic polymers have long been employed as insulating materials in power transmission cables, pulse generators, high-voltage motors, and transformers [3]. Their outstanding inherent properties, such as good processability, high breakdown strength, and excellent flexibility, make them suitable for these applications [4]. Breakdown strength is termed as the maximum voltage of an insulating material can withstand before the collapse of insulation system and expressed as Volts per unit thickness. It measures the dielectric strength of an insulator, so the higher the voltage value of a material, the greater its performance as an insulator [5].

**Fig. 1:**
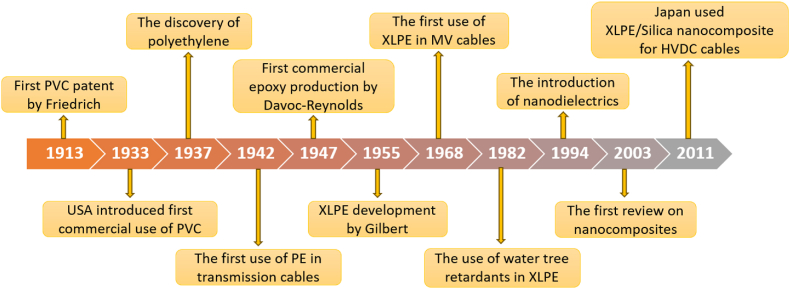
The development of insulation cables throughout the history.

Cross-linked polyethylene (XLPE) insulation was introduced in 1963 by General Electric Company. Meanwhile, preferred insulating materials are based on synthetic polymers like polyvinyl chloride (PVC) due to their affordability, durability, hardness, and superior tensile strength. However, XLPE surpasses them in terms of dielectric permittivity (also known as dielectric constant), loss factor, dimensional stability, and solvent resistance. Moreover, it can operate at higher temperatures compared to its other derivatives. The major hindrance faced by XLPE insulation in high voltage applications is not that different from other dielectric materials, which is space charge accumulation. Unlike rubber, PVC, and impregnated paper cables, XLPE consists of crosslinking by-products that initiate space charges caused by ionisation. Secondly, unlike rubber and PVC that exhibit greater flexibility and resilience, XLPE insulation is susceptible to brittleness in low temperatures, posing challenges for applications in cold climates. Fig. 1 outlines the development of insulation cables since 1913.

## 2 Cross-linked polyethylene

**Fig. 2:**
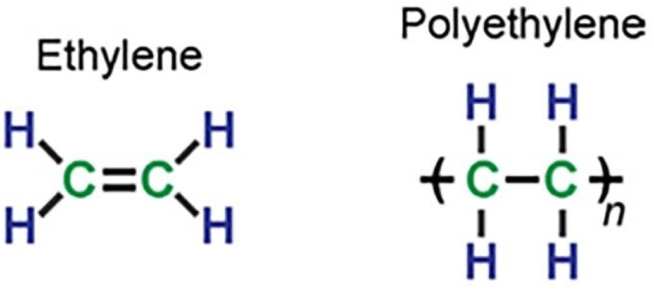
The chemical structure of ethylene and polyethylene [11].

Polyethylene (PE) is a type of polyolefin composed of repeating ethylene unit (CH_2_=CH_2_). Its versatile properties of being lightweight, durable, thermoplastic, having low moisture absorption, good chemical resistance, and electrical insulation popularize its application in both packaging and non-packaging plastics. It is available in various forms such as branched PE, low-density PE (LDPE), linear PE, high-density PE (HDPE), and linear low-density PE (LLDPE). Fig. 2 illustrates the chemical structure of ethylene and polyethylene. Basically, cross-linking process changed thermoplastic PE into thermoset XLPE through the establishment of three-dimensional network structure. Consequently, chemical and physical properties are enhanced, resulting in improved impact strength, creep resistance, temperature resistance, and environmental stress crack resistance. The cross-linking process is categorised into chemical and physical methods. The chemical method uses supplementary compound, such as organic peroxide-based and silane-based substances to generate free radicals. The physical method utilizes high-energy radiations like gamma rays or ultraviolet (UV) radiation to initiate cross-linking with adjacent polymer chains. Table 1 provides further details on the chemical and physical cross-linking processes. Certainly, each process has its own merits and demerits. Therefore, the cross-linking method must be selected by taking into account intended applications/products, operational cost, gel content, and possible hazards and risks associated with the materials.

**Table 1:** The cross-linking methods of XLPE [12].

	Chemical		Physical
	Organic peroxide	Organic silane	Electron irradiation
Materials	• Dicumyl peroxide	• Vinyltrimethoxysilane • Dicumyl peroxide • Antioxidant • Crosslinking catalyst (dibultinlaurate)	• Ketone-based photo initiators, such as benzophenone or benzyl dimethyl ketal
Mechanism	• Organic peroxide acts as an initiator to generate free radicals	• Reactive silane molecules are graft to PE backbone	• High-energy radiations liberate free radicals
Process	• Four manufacturing processes namely Engel, Dioplast, Pont a’ Mousson and Ultra High Frequency Initiation (UHFI) - Engel process blends together PE, peroxide and stabilisers under high pressure - Dioplast process extruded PE into a media containing peroxide. High temperature and pressure decomposed the peroxide and initiated cross-linking - Pont a’ Mousson process extruded PE and peroxide and then cross-link in a salt bath at 250–280 °C - UHFI used microwave radiation to decompose the peroxide • Operating pressure at 15–20 kg/cm2 • Extrusion - Operating temperature at 250–350 °C - Operating pressure at 15–20 kg/cm2	• Two manufacturing processes namely Monosil and Sioplas • Monosil is a single-step process - All materials are mixed together with PE during conventional extrusion process • Sioplas is a two-step process - First step is preparing silane grafted PE (part A) and catalyst masterbatch (part B) - Second step is blending part A with B • Curing - Water or steam temperature around 70–90 °C	• Exposure to high-energy radiations like beta rays, gamma rays, or UV radiation
Advantages	• High degree of cross-linking up to 90 % • No restriction on material thickness (applicable for LV–HV cables)	• Monosil - Cost effective on large production - Process flexibility • Sioplas - Wide range of applications - Can incorporate reinforcements	• Controllable irradiation doses • Can impart sterilization
Disadvantages	• High processing temperature • Longer processing time	• High energy consumption using water or steam • Slow production time due to curing - Risk of moisture diffusion during curing	• High cost of equipment (accelerator) • Restricted material thickness of 1–2 mm
Final properties	• The formation of C–C cross links provides superior thermal stability, mechanical strength, chemical resistance and electrical insulation properties over silane XLPE • The elongation at break, yield strength and tensile at break deteriorate as peroxide content increased.	• The formation Si–*O*–Si cross links is weaker compared to C–C bonds, though it still provides good mechanical strength and chemical resistance • Mechanical properties improve with increasing cross-linking time.	• Heightened thermal stability and resistance against thermal degradation over peroxide XLPE. • Young's modulus increases as radiation dose increase

**Fig. 3:**
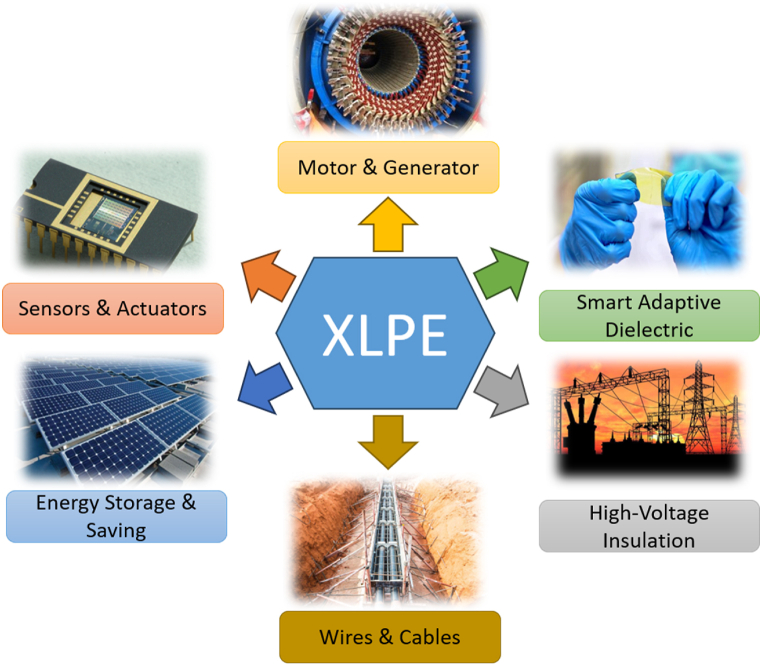
Typical applications of XLPE-based polymers in electrical components and systems.

In the case of cable insulation, cross-linking is primarily aimed at enhancing the material's thermal stability under load, ensuring that functional properties are preserved even at high temperatures and reducing shrinkage. This superiority placed XLPE ahead of PVC as an insulation material for conductors, since XLPE does not shrink when exposed to conductor-generated heat. Currently, XLPE is the most widely used polymer for insulation material in alternating current (AC) transmission lines within public and industrial power systems [6,7]. Besides exceptional insulating capabilities, it is essential for a dielectric to possess low dielectric loss, high dielectric constant, high thermal conductivity, enhanced thermal stability, and superior resistance against electrical stress, tracking, and weathering [[8], [9], [10]]. The ubiquity of polymeric materials in electrical-based apparatuses, cable, and tools drives the need for even slight improvement in dielectric performance to compete and thrive in the industry. Fig. 3 indicates several applications of XLPE in electrical components and systems.

## 3 Modification of XLPE

There are several methods to fabricate modified XLPE, with the most established ones being melt-blending, solution casting, in situ polymerisation, and sol-gel processing [1], [13]. Each additive used in the process has its own unique features and physicochemical properties that will influence the credibility of the final product. Achieving good compatibility is of great importance in order to effectively incorporate these exceptional properties into XLPE. In particular, nanoparticles

Require additional processes to increase repulsive forces and reduce gravitational forces between particles. Smaller molecular compounds such as antioxidants and voltage stabilisers can migrate and precipitate on the surface if stabilised chemical bonds are not established with their respective matrix.

Direct mixing includes two approaches: melt-blending and solution casting. In the melt-blending approach, the polymer is melted into a molten state before being mixed with filler under high pressure and temperature. Solution casting uses a solvent to dissolve the polymer, ensuring consistent filler dispersion, followed by the evaporation of the solvent. In situ polymerisation involves the direct mixing of liquid monomer resin and filler, followed by polymerisation initiated by heat, light, or curing agents. The sol-gel process, on the other hand, is a wet chemical technique where the colloidal suspension of the filler forms sols, which undergo hydrolysis, condensation, gel formation, aging, solvent evaporation/calcination, and ultimately crystallisation [14]. During the preparation of polymer composites, agglomeration of fillers can occur due to filler-filler clustering and filler-matrix incompatibility. Surface modification of the fillers prior to the mixing process can address this compatibility issue [15]. There are three commonly employed approaches to enhance the interfacial adhesion bond between the filler and matrix: chemical treatment, grafting, and physical adsorption of polymers. Chemical treatment involves the use of organic silane to modify the filler surface by introducing functional groups that establish strong chemical bonds with the polymer matrix [16]. Grafting refers to attaching polymer functional groups onto the filler, reducing filler-filler interactions. Physical adsorption, on the other hand, involves the adsorption of polymer dispersants onto the filler surface to form non-covalent bonds. Unlike chemical bonds, physical bonds are weak and reversible, which relies on van der Waals force, hydrogen bonding and other relatively weak interactions.

## 4 The importance of XLPE modification in power transmission cables

In actual power transmission systems, the dielectric insulator is continuously subjected to electrical and thermal stresses. The deterioration of insulating performance can easily occur under high voltage and temperature conditions [17]. At full load operation of high-voltage transmission, the emission of excessive hot electrons tends to accumulate within the insulator, forming space charges. This accumulation can result in partial discharge (PD), which is a phenomenon characterized by localized electrical discharges within the insulation system. These discharges can eventually lead to insulation failure [10], [18]. The main function of power transmission cables is transporting and distributing electrical energy over long distances. Therefore, the superior dielectric properties of the insulating material are prioritized over other requirements such as flexibility and thermal expansion to keep the reliable operation of the system. Dielectric strength plays a pivotal role in electrical insulation in high voltage applications, which considered invaluable in electrical industry. The dielectric properties dictate the capacity of the insulation material to withstand high electric fields without suffering breakdown, thus securing the integrity and dependability of the electrical system. In this regard, the selection of a material with distinguished intrinsic properties like XLPE is preferred over materials such as ethylene propylene rubber (EPR) and silicone rubber (SiR). While EPR and SiR offer high flexibility, they have higher dielectric loss compared to XLPE [19]. Contrarily to dielectric constant, dissipation factor, which is significantly affected by temperature and electric stress, contributes to the increase in dielectric loss and imposes a serious limitation on power transmission performance [20]. In contrast, XLPE can withstand high temperatures while maintaining its electrical properties. However, XLPE still faces critical consequences due to the occurrence of space charges and stress inversion. The accumulation of space charges in any transmission cable distorts the electric field and can lead to the breakdown of the power transmission system [21]. Space charge phenomenon can be caused by factors such as heterogenous conductivity and permittivity of the insulator material, charge injection via electrodes, and ionisation of impurities (antioxidants, crosslinking by-products, dissociable chemicals) under DC voltage [1]. High temperature gradients can induce stress inversion, resulting in a higher electric field at the outer semiconductor screen compared to the inner one adjacent to the conductor [22]. The electric field generated along the cable radius is heterogenous, creating thermal gradients due to losses of circulating current [23]. The resistivity of the insulator is more dependent on electric field and temperature than permittivity [24]. To enhance the reliability of insulating materials, new approaches are essential to address these drawbacks. Nowadays, a wide variety of additives have been implemented to mitigate these issues. Basically, modifications can provide either advantages or disadvantages to the end product. The unique characteristics of additives can introduce newly developed products.

**Fig. 4:**
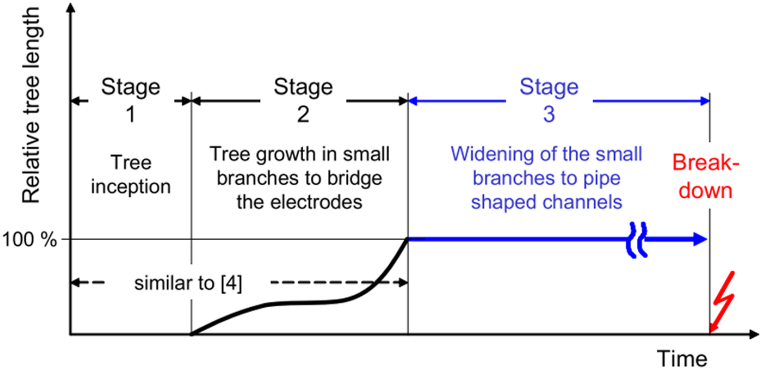
The tree propagation stages until breakdown occurs [35].

In the context of defects and failures in the insulation system, additives also play a crucial role. Defects and failures in the insulation system are difficult to predict and require significant costs and efforts to be resolved [25]. When the supplied voltage along the conductor can no longer be afforded in a stable state, electrical breakdown occurs, causing a power transmission failure. Usually, electrical breakdown is related to structural defects in the material and its inability to withstand high voltage stress. For example, PD, is caused by the presence of defects or void areas in the insulating material. This phenomenon increases the likelihood of water and electrical treeing propagation. Fig. 4 represents the tree propagation stages before experiencing final breakdown. Nanoparticles offer advantages to XLPE nanocomposites by acting as nucleating agents [26], establishing conductivity networks [27], scattering high-energy electrons [28], and suppressing PD [29]. By occupying free volume within the matrix, the probability of PD is reduced, and local electric field decreases due to differences in electrical permittivity [14]. Voltage stabilisers, small-molecular-weight substances with high conductivity, can migrate to the defect regions of polymer to reduce the electric field [30]. Also, they can attenuate the high-energy electrons to weaken their impact on polymer scission. On that account, the term “prevention is better than cure” remarkably fits the need of refining XLPE to be applied in power transmission cables.

Breakdown voltages play a vital role in the advancement and utilisation of nanocomposites for electrical insulation. There are different types of break down voltage, including AC, DC, and impulse [31], [32], [33]. Equation (1) is the expression for the breakdown strength (E) of a dielectric material [34]:

(1) $$E=\frac{V}{d}$$

Where, V is breakdown voltage and d is dielectric material thickness.

## 5 Nanofillers reinforced XLPE nanocomposites

The introduction of nanoparticles, including metals, non-metals, and ceramics, into insulator materials is a widely used approach to address charge accumulation issues. There are four types of nanofillers based on their dimensional geometries. Zero-dimensional nanomaterials refer to materials with all three dimensions in the nanometric scale, such as spherical silica particles. One-dimensional nanomaterials have one dimension outside the nanometric scale and exhibit elongated structures, such as carbon nanotubes or cellulose whiskers. Two-dimensional nanomaterials possess only one dimension in the nanometric scale and have plate-like shapes, such as nanocoating, nanofilms, and nanolayers. Meanwhile, three-dimensional nanomaterials consist of clusters of nano-sized crystals, with all dimensions exceeding the nanometric scale, such as diamond and graphite.

The properties of nanocomposites are significantly influenced by the dispersion of nanofillers within the polymer matrix. Nanofillers tend to aggregate during the fabrication process, which can deteriorate the mechanical, electrical, and chemical properties of the final product. The electrostatic force responsible for holding nanoparticles together is a weak interaction. Therefore, processing methods for nanocomposites apply strong forces to disrupt the agglomeration of nanoparticles and disperse them as individual particles. One commonly used method is the melt mixing method, which utilizes high shear forces to break down the aggregation of nanoparticles and prevent reaggregation during mixing. Another used method is the solution mixing method, where nanofillers are dispersed in a solvent solution in which the polymer is also soluble. This method usually involves auxiliary processes such as sonication or ultrasonication to agitate the nanofiller bundles. The high-frequency waves generated during sonication provide higher energy than the interaction energy between the nanofillers, facilitating their dispersion. Another method is surface modification, which has been mentioned in section 3.0. Surface modification is performed prior to the mixing process. Surface modification of inorganic nanofillers (aluminium oxide, silica oxide, titanium oxide and zinc oxide) goes through a silanization process to graft hydroxyl groups onto their surfaces. On the other hand, carbon-based nanofillers, like carbon black, carbon nanotubes, fullerenes, and graphene, undergo Diels-Alder reactions to modify their surface properties.

### 5.1 Metal oxide

Zinc oxide (ZnO) is widely recognized as an excellent non-linear semiconductor material, widely used in the rubber industry as a vulcanization catalyst and to enhance thermal conductivity [36]. Its high thermal conductivity and non-linear characteristics make it particularly useful for electric stress control. The non-linear conductivity of ZnO, caused by grain boundary effects, defect states, and polarisation mechanisms are responsible for improving the dielectric performance of nanocomposites [[37], [38], [39]]. This non-linear current-voltage (I–V) behaviour caused by potential barriers at grain boundaries, intrinsic defects such as oxygen vacancies, and synergetic surface state interactions in nanostructured ZnO [40], [41]. These factors allow ZnO to have higher breakdown strength by uniformly distributing electric fields, increasing energy storage capacity through increased permittivity and additional polarisation mechanisms, and lowering dielectric losses through interface effects that trap charge carriers. Furthermore, ZnO's thermal stability helps to maintain consistent dielectric properties across a wide temperature range, making ZnO-based nanocomposites [42]. Mansor et al. [43] discovered that the addition of ZnO to XLPE induced carrier traps that suppress space charge, leading to a higher critical electrical field for the formation of percolation paths. This was evident in the improved electrical tree inception voltage, which increased from 15.09 kV (unfilled) to 17.30 kV (1 % ZnO). Similarly, Sharshir et al. [44] pointed out that the incorporation of 1 % ZnO into XLPE resulted in the lowest AC conductivity due to the insufficient mass fraction to reach the percolation threshold. Exposure to high-energy electrons emitted from electron beam irradiation empower electrons hopping process between ZnO nanoparticles. At low loading levels, the nanocomposites exhibit high electrical resistivity without showing non-linear characteristics, unless a sufficient amount of ZnO is present. Scaling up the loading causes a decrement in resistivity. The non-linear conductance coefficient of ZnO-based nanocomposites is influenced by specimen geometry, operating temperature, and pressure. The coefficient increases significantly with temperature and decreases with pressure. Additionally, the disparity in chemistry and physics between the filler and the polymer creates a low-resistivity charge interface region. Consequently, charges generated under an applied electric field flow into this region and are impeded to a certain extent, hindering the formation of a conductive path. Dong et al. [36] reported that the XLPE nanocomposites with a filler loading of 15 % exhibited the best characteristics of non-linear conductivity, but this non-linear behavior could only be observed when the applied electric field exceeded 12 kV/mm. The decrease in resistivity indicates that the nanofiller loading met the threshold for non-linearity.

**Fig. 5:**
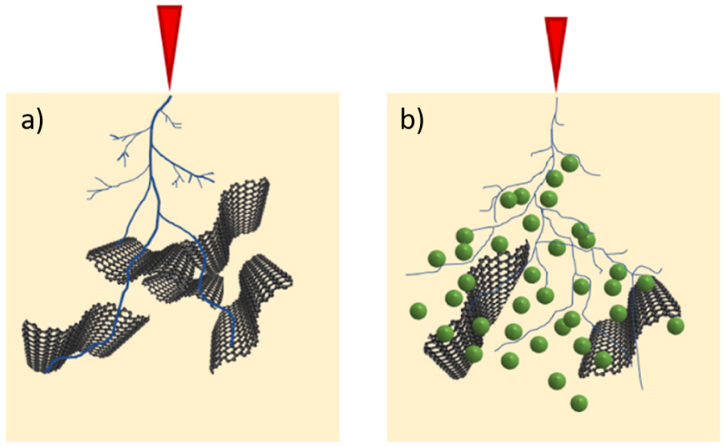
The illustration of electrical tree propagation in the a) SiR/graphene and b) SiR/graphene/Al2O3 [49].

Aluminium oxide (Al2O3) is another commonly used filler due to its low cost, relatively high thermal conductivity, and electrical resistivity. It has been observed that the addition of Al2O3 to XLPE can reduce the ignition and heat release rates [45]. In a study by Mohamed et al. [46], the reinforcement of Al2O3 in XLPE resulted in an increase in the electrical tree inception voltage value from 11.2 kV (unfilled) to 14.8 kV (1 %). The time required for treeing to reach 2 mm also increased from 21 h (unfilled) to 23 h (1 %), and the insulation breakdown time increased from 23 h (unfilled) to 26 h (1 %). Al2O3 has also been incorporated into silicone anti-failure rejuvenation fluid to restore water trees in aged medium voltage cables. This restoration process involves the use of a silane coupling agent to react with water and form silanol molecules, which then form siloxane and hydrogen bonds through interactions with hydroxyl groups on the nanofiller. Oxygen and carboxyl groups residing in the micro voids of the water tree reacted with phenyl and methyl groups of the silane. Consequently, silane-treated Al2O3 is more easily bonded with XLPE. When using this approach in Refs. [47], [48], a gradual decrease in the dielectric loss factor of the injected aged cables was reported compared to new unused and aged cables. On the other hand, Yang et al. [49] reported that the hybridisation of Al2O3 with SiR/graphene partially isolates graphene particles, preventing the establishment of conductive pathways and increasing the threshold field strength required to enter a non-linear state. Moreover, the charge conduction across the Al2O3 particle surface is impeded, significantly influencing the obstruction of the electrical tree channel. Fig. 5(a) and (b) show the illustration of electrical tree propagation in the SiR/graphene nanocomposites with and without Al2O3, respectively. Additionally, Ghezelbash et al. [50] and Yang et al. [51] highlighted the thermal stabilisation effect of Al2O3, which enhances the high-temperature dielectric performance of polyimide nanocomposites.

The density of trap-states Nt is an essential parameter for understanding charge transport in nanocomposites. It can be represented as Equation (2) [52]:

(2) $$N_{t}=\frac{2EE_{0}V_{TFL}}{eL^{2}}$$

Where, ℰ and $E_{0}$ are the dielectric constants of nanocomposite and vacuum, $V_{TFL}$ is the trap-filled limit voltage, e is the elementary charge (approximately 1.602 × 10–19 coulombs), and L is the thickness of the material.

### 5.2 Ceramic

Titanium oxide (TiO2) is a commonly used reinforcing material to improve polymers in electrical and thermal insulation applications due to its high relative permittivity, which can reach levels as high as 100 [14]. Although different forms of TiO2 have the same chemical formula, they exhibit distinct structures and phases, leading to diverse chemical properties and crystalline structures. In Ref. [53], it was determined that the optimum loading of TiO2 was 5 % in maximizing AC breakdown strength at room temperature, elevated temperature (250 °C), and after subjected to thermal aging. The practicability of using a high loading can be associated with the low surface energy of TiO2 and the incorporation of dicumyl peroxide (DCP), which prevents the nanoparticles from clustering together.

In dielectric solids with glassy and crystalline structures, thermal conduction occurs via elastic vibrations of the lattice, known as phonons. The integration of nanofillers with high thermal conductivity facilitates efficient heat transfer, as heat can swiftly propagate along these materials with elevated thermal conductivity. However, the introduction of nanofillers can also increase thermal resistance due to two factors: disparities in phonon spectra across phases and scattering at the interfaces between these phases [54]. These phenomena coexist and interact with each other. Additionally, the existence of additional phases can alter the physical properties of the matrix, thereby influencing its thermal conductivity of the matrix. In case of low nanofiller content, particles are dispersed and isolated within the matrix. In this scenario, the thermal interface resistance or boundary scattering between fillers and the matrix plays a crucial role in determining the thermal conductivity of nanocomposites. In a study by Said et al. [55], AC breakdown strength reached the highest value at 2 % TiO2 loading when functionalised by amino silane. In the case of Rahman et al. [21], it was observed that TiO2 loading exceeding 1 % started to decline AC breakdown strength of the XLPE nanocomposites. In the absence of surface modifier, notable inferiority was observed in breakdown strength value and dispersion of nanofiller. Their large surface area also has a great tendency to stimulate agglomeration.

**Fig. 6:**
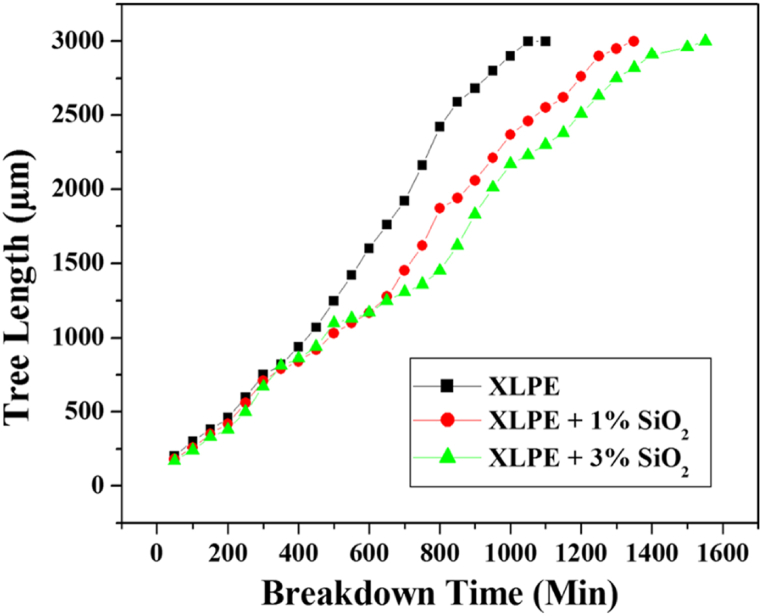
The electrical tree growth against time [56].

**Fig. 7:**
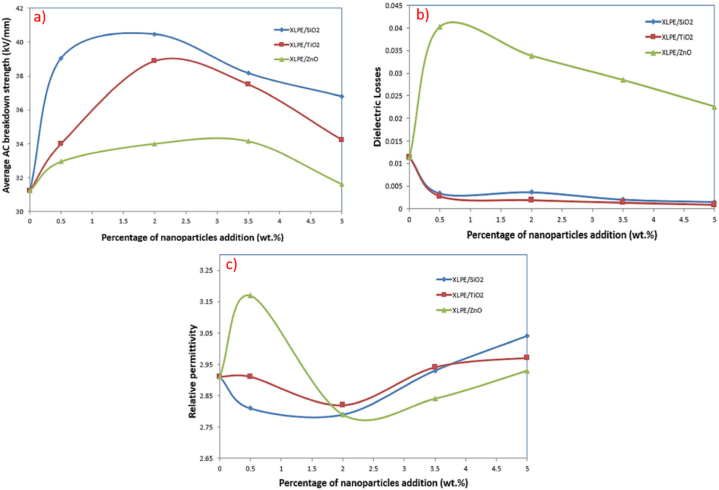
a) The average AC breakdown b) dielectric loss and c) relative permittivity of XLPE nanocomposites at different loading of SiO2, TiO2 and ZnO [59].

Silicon dioxide or silica (SiO2), in the form of crystalline and fumed (amorphous), is widely used in the manufacturing of materials for electronic appliances. It has high electrical resistivity, low dielectric constant and thermal conductivity. Owing to these features, silica-based nanocomposites always end up with the lowest thermal conductivity compared to other nanofillers. Kalaivanan & Chandrasekar [56] disclosed that the addition of SiO2 into XLPE extended the breakdown time due to electrical treeing. Specifically, the breakdown time increased to 18 h for 1 % SiO2 loading and 21 h for 3 % SiO2 loading. XLPE nanocomposites recorded higher electrical resistivity than pristine XLPE validating SiO2 attribute is beneficially contribute to the longevity of insulation material. As observed in Fig. 6, higher loading of SiO2 required longer time for electrical tree to propagate. Similarly, it was observed by Purushotham et al. [57] that the introduction of SiO2 acted as a barrier to detain the electrons generated during high electric field stress, slowing down the propagation of the electrical tree growth that led toward dielectric breakdown. In comparison with pristine XLPE, SiO2 loading increased the breakdown time from 450 min to 580 min for 1 %, 650 min for 3 % and 670 min for 5 %. The presence of nanofillers also induced trap areas that capture the charge carriers hindering the pathway of treeing. Sharad and Kumar [58] disclosed that surface modified SiO2 positively enhanced the nanofiller dispersion. At 3 % loading, the discharge inception voltage and breakdown voltage values of unmodified XLPE/SiO2 are 7.8 kV and 35.6 kV, respectively meanwhile modified XLPE/SiO2 are 10.1 kV and 40.5 kV, respectively. The surface modification using amino silane promotes fine dispersion of SiO2 to form large interfacial areas, which helpful in suppressing XLPE degradation by rising inception voltage. Theoretically, higher inception voltage indicates superior insulating properties as it requires higher voltage level to initiate PD. In a similar set up, Said et al. [59] discovered that at 2 % loading of modified SiO2, XLPE nanocomposites show most relevant dielectric performance with highest AC breakdown voltage, lowest relative permittivity similar to XLPE/ZnO and dielectric loss comparable to XLPE/TiO2. Fig. 7(a), (b) and 7(c) demonstrate the average AC breakdown, dielectric loss and relative permittivity of XLPE nanocomposites at various loading of SiO2, TiO2 and ZnO, respectively. It is also important to note that a large contrast of dielectric constant between nanofillers and polymer matrix triggers the distortion of local electric field subsequently lowering the breakdown strength of nanocomposites. Contrariwise, unmodified SiO2 tends to agglomerate consequently hasten PD to reach towards the ground electrode. The agglomeration of unmodified SiO2 leads to the formation of localised regions with high electric fields and voids, which act as sites for the initiation of PD [60], [61]. This PD activity results in localised heating and erosion, which speeds up insulation degradation and may lead to early insulation breakdown.

Another interesting Si-based nanofiller is silica carbide (SiC), which is characterized by high thermal conductivity and high temperature stability. Similar to ZnO, its non-linear electrical conductivity aspect is advantageous for stress control in generator end windings, cable, and HV accessories [54]. Zhang et al. [62] stated that the expansion of space charge in XLPE/SiC nanocomposites can be suppressed owing to the unsaturated electronic structure and magnetism on the Si- or C-terminated surface of SiC, capturing the emitted hot electrons. High-energy electrons are neutralised by the SiC distribution, which suppress charge accumulation and ultimately preventing XLPE from breakdown.

**Fig. 8:**
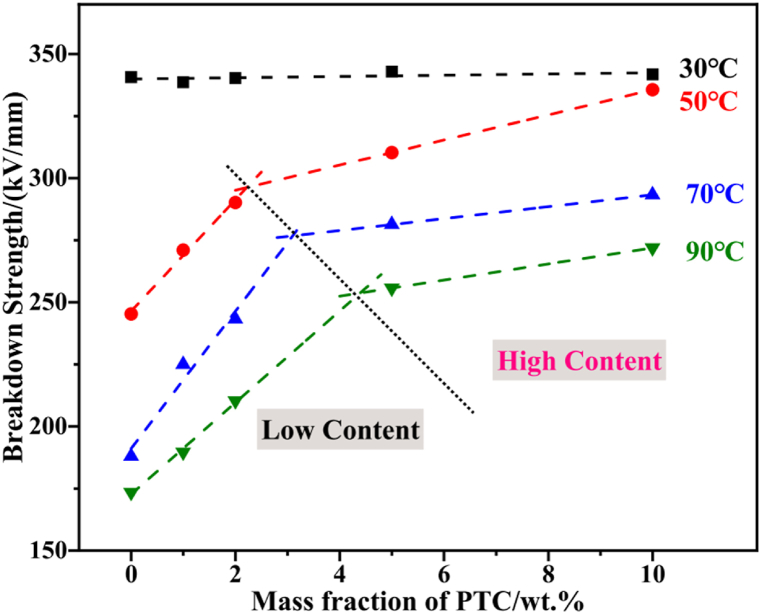
The breakdown strength of XLPE at different loading of positive temperature coefficient (BaTiO3) [63].

Barium titanate (BaTiO3) is ceramic material possessing a high dielectric constant and low dielectric loss, commonly used as a filler to improve dielectric constant, power storage, mechanical, and thermodynamic properties of polymer nanocomposites. In a study by Jiang et al. [63], silane-modified BaTiO3 was mixed prior to the crosslinking of XLPE. The distribution of BaTiO3 restricted the electron movement when temperature was elevated subsequently limiting the kinetic energy impact towards the XLPE molecular chain. Correspondingly, active charge carriers were restrained by dense deep trap, henceforth, the electrical resistivity and breakdown strength of nanocomposites were heightened. Nonetheless, the enhancement effect on the breakdown strength worsened as nanofiller loading exceed optimum amount of 5 % as indicated by Fig. 8. XLPE/BaTiO3 nanocomposites exhibit low electric field distribution owing to its high dielectric constant and weak dependence of dielectric loss temperature ascribed to restricted poly chain mobility. Therefore, electrical resistivity that depend on electric field and temperature indicated small and distinct improvement with increment of nanofiller loading and temperature. Boron nitride (BN) is known to possess high temperature resistance, high thermal conductivity, high electrical resistivity, low dielectric constant and low density suitable for high conductive composite application. Boron nitride nanosheets (BNNSs) offer advantages over conventional BN due to their layered structure similar to graphite. Zhou et al. [64] found out that the uneven size of BNNS formed larger interfacial bonds with XLPE to generate more traps and minimise electrical field. The breakdown strength signifies a notable difference between XLPE/BN (349.2 kV/mm) and XLPE/BNNS (403.8 kV/mm). On another aspect, the compactness or crystalline structure plays an important role in holding the mobility of charge carriers. With limited site for polymeric molecular chains to interact, higher loading of nanofiller led to repulsive action of XLPE thus interaction energy weakens leaving large free volume to conduct charges.

### 5.3 Non-metal

Under long-term operation, the insulator is subjected to thermal oxidation, chain scission, and change in crystallinity [65] [66], [67]. The trap density and trap energy level play a significant role in the breakdown mechanism. At elevated temperature, the increased thermal conductivity facilitates the mobility and density of the charge carriers. Additionally, the dominant carbonyl groups intensify the density of shallow traps replacing the deep traps. High-energy electrons have higher tendency to escape shallow traps compared to deep traps. A limitation of inorganic nanoparticle is that it requires high loading to manifest the functional properties causing dielectric to become heavier, which restrict its industrial applications.

An ideal dielectric should consist of less nanoparticle loading but exhibits good electrical performance. Graphene is a unique two-dimensional sheet of sp2-hybridized carbon structure with large specific surface area can generate large number of interfacial regions even at very little loading. Li et al. [68] revealed that graphene dispersed prior to crosslinking process of XLPE yield insignificant change in conductivity owing to the fine dispersion and large adjacent distance between nanofillers. However, higher loading initiates overlapping between interaction sites of graphene-matrix, creating paths for electrons between molecular chains to accumulate space charge. Du et al. [69] identified that the threshold loading of graphene oxide (GO) into XLPE is 0.01 % since higher loading will constructs conductive channels accelerating the charge carrier movement. At elevated temperature, additional 0.1 % loading of GO trigger thermal ionisation of impurities and defects, weakening the role of the nanoparticle-matrix interaction phases to capture charge carriers and restrain their movement. Meanwhile, by adding reduced graphene oxide (rGO), Wu et al. [70] pointed out that the breakdown voltage of XLPE/rGO films were measured 22.75 kV/mm at 2 % loading and 1.9 kV/mm at 3 % loading. At low loading, a non-percolative structure of nanocomposites are established with wide gap width between rGO thus obstructing the initiation of conductive network for the electrical percolation. rGO is a derivative of GO, typically processed chemically and thermally in order to minimise oxygen content of unstable GO. In its oxidised form, abundant oxygen functional groups of GO have lower electrical conductivity than rGO. The reduction process restores double-bonded aromatic carbon atoms thus inducing conductivity by multiple times [61]. In term of their compatibility with XLPE, rGO slightly have better compatibility over GO, attributed to lesser oxygen functional groups rendering it less polar. Hence, it can be dispersed better in non-polar polymers compared to GO. The apparent difference between GO and rGO loadings are associated with their respective surface area.

**Fig 9:**
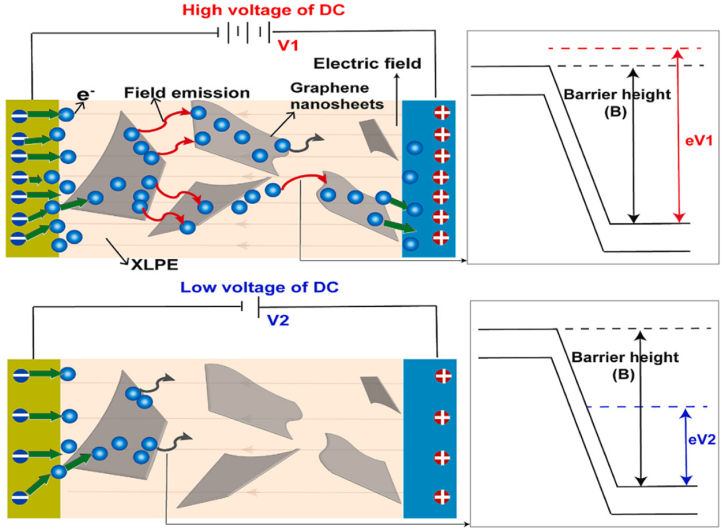
The schematic diagram of electron movement in respect of voltage applied [70].

Fig. 9 illustrates the electron movement in respect of voltage applied. High voltage is compulsory for the electron to overcome the energy barrier generated by the wide gap width of rGO. Basically, the tortuous path established by finely dispersed nanofillers elevates the critical electrical field for overall breakdown to trigger. When encountered nanoparticles, the breakdown propagation is obstructed into a twisted path around the matrix/filler interface. In the case of higher aspect ratio nanofillers such as BNNS and graphene, the breakdown path is prone to penetrate through it. As a result, the breakdown propagation required a critical value of applied electric field to advance. The aspect ratio and dispersion of nanofiller are vital influences to design nanocomposites with superior breakdown strength, enhance dielectric constant and boosted energy density [71]. Table 2 indicates the direct and alternating current breakdown strength of XLPE doped with various nanofillers.

**Table 2:**
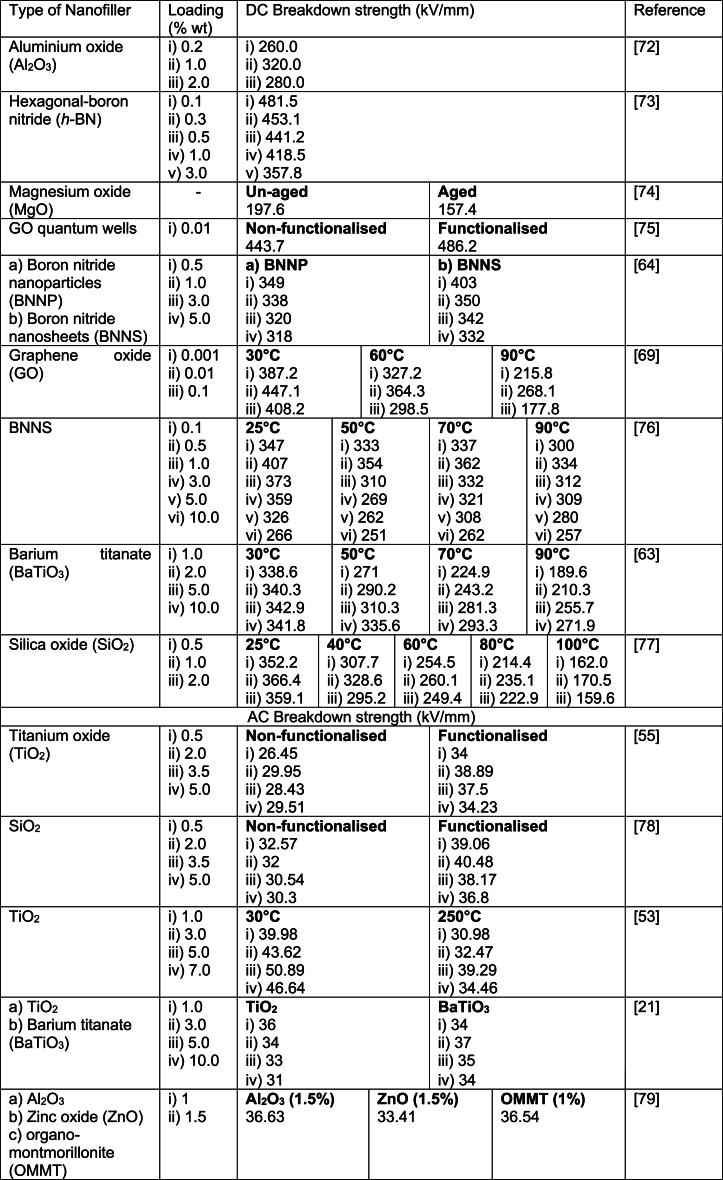
The dielectric breakdown strength of various nanofillers reinforced XLPE [[21], [53], [55], [63], [64], [69], [72], [73], [74], [75], [76], [77], [78], [79]]

## 6 Voltage stabiliser incorporated XLPE

**Fig. 10:**
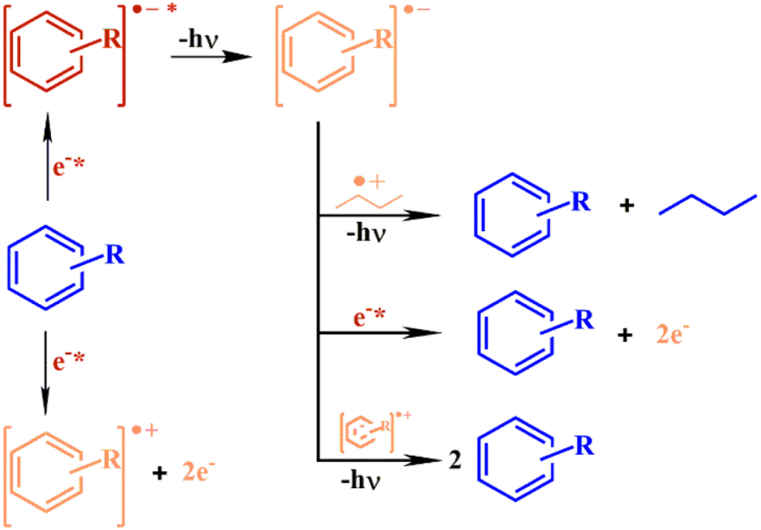
The mechanism of voltage stabiliser in capturing high-energy electrons [6].

The presence of polar groups and conjugated double bonds in voltage stabilisers can initiate deep traps that supress electric charge carriers in the polymeric material system. The effectiveness of voltage stabiliser greatly depends on its electron affinity, where high efficiency voltage stabiliser is likely to have high electron affinity, low ionisation potential and narrow energy gap. The grafted polar groups act as a scavenger that absorb and consume energy of high-energy electrons inhibiting their impact on the macromolecules. When high-energy electrons are attenuated, the macromolecule degradation is suppressed and extending life time of a dielectric material. Fig. 10 reveals the interaction between high-energy electron with voltage stabiliser to dissipate energy by producing anions and cation radicals. Dong et al. [80] observed an increment in AC breakdown strength from 134.4 kV/mm to 155.3 kV/mm and tree initiation voltage (TIV) from 5.702 kV to 7.446 kV by grafting 1 % of 1-(4-vinyloxy) phenylethenone (VPE) onto XLPE. This can be associated with VPE ability to absorb high energy electrons and dissipate it via exothermic process, which restricted their collision onto polymer chains. After subjected to thermal ageing at 90 °C for six days, the stabilised XLPE/VPE showed lower reduction in AC breakdown strength from 155.3 kV to 122.8 kV compared to XLPE/acetophenone (AP) from 158.7 kV to 138.5 kV. Furthermore, grafting during crosslinking process minimises tendency of migration during thermal ageing, which verified by the existence of VPE absorption peak by FTIR analysis.

On the other hand, Zhang et al. [81] introduced 2-(4-benzoyl-3-hydroxyphenoxy) ethyl acrylate (BHEA) during the melting blending process prior to the crosslinking process and noted a slight improvement in heat-resisting capabilities of XLPE, as observed in the minor increment of DC breakdown strength at higher temperatures. However, at room temperature, pristine XLPE excelled XLPE/BHEA composites. Polar functional groups of BHEA are unfavourable for the crystallisation inducing extra free volume to form loose molecular structure, which permitted higher electron mobility. Even though, the increment in temperature catalysed charge carrier mobility, BHEA plays the role to capture and scatter the energised charge carrier. Also, x-ray photoelectron spectroscopy (XPS) analysis confirmed that the crosslinking reaction mitigates the extraction and migration of the BHEA molecules from the XLPE/BHEA composites. Deng et al. [82] grafted 4-(4-benzoyl-3-hydroxyphenoxy)-4-oxobut-2-enoic acid (MDVS) onto XLPE, which result in the improvement of DC breakdown strength from 330.8 kV/mm to 352.3 kV/mm. In addition of retarding polymer chain scission, grafting of MDVS promotes heterogeneous nucleation yielding slight boost in the crystallinity, crystallisation and melting temperature. As space charge is often initiated within defect region, improvement in these three factors signify highly organised structure of molecules. Higher crystallisation temperature means slower crystallisation and higher crystallinity, which require significant amount of energy (in the form of heat) to destroy the crystal structure. Since a well structural order is formed, higher electrical field is required to damage the insulator and propagate electrical tree growth.

**Fig. 11:**
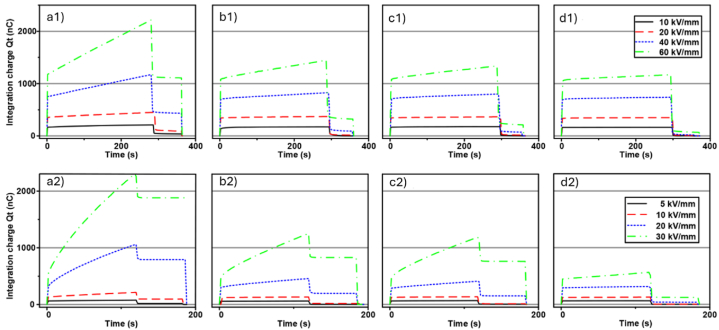
The space charge behaviours of XLPE doped with polycyclic aromatic compounds under various electric field and temperatures; a1) XLPE, b1) XLPE/PAC-A, c1) XLPE/PAC-B, d1) XLPE/PAC-C at 25°C and a2) XLPE, b2) XLPE/PAC-A, c2) XLPE/PAC-B, d2) XLPE/PAC-C at 80°C [83].

**Fig. 12:**
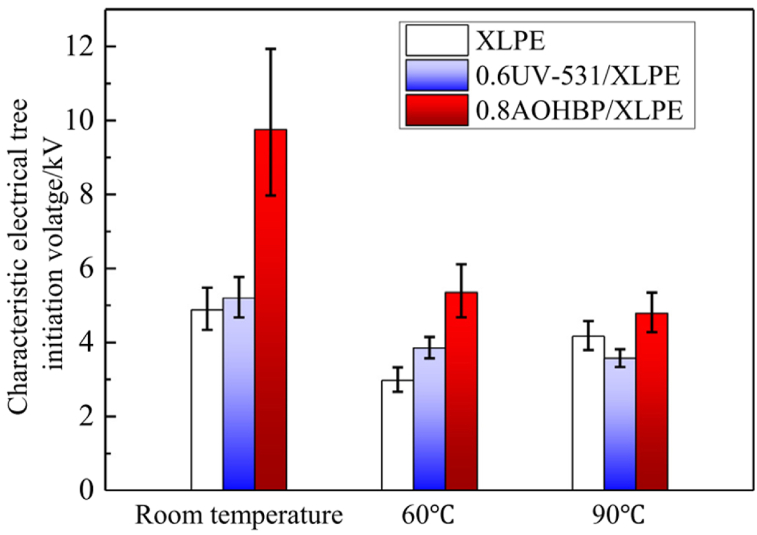
The electrical tree initiation voltage of XLPE and its composites under different temperatures [85].

**Fig. 13:**
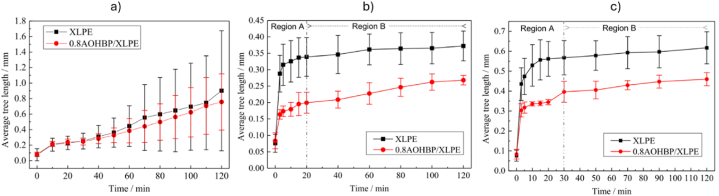
The electrical tree propagation stages of XLPE and its composites under different temperatures; a) room temperature, b) 60°C, and c) 90°C [85].

**Fig. 14:**
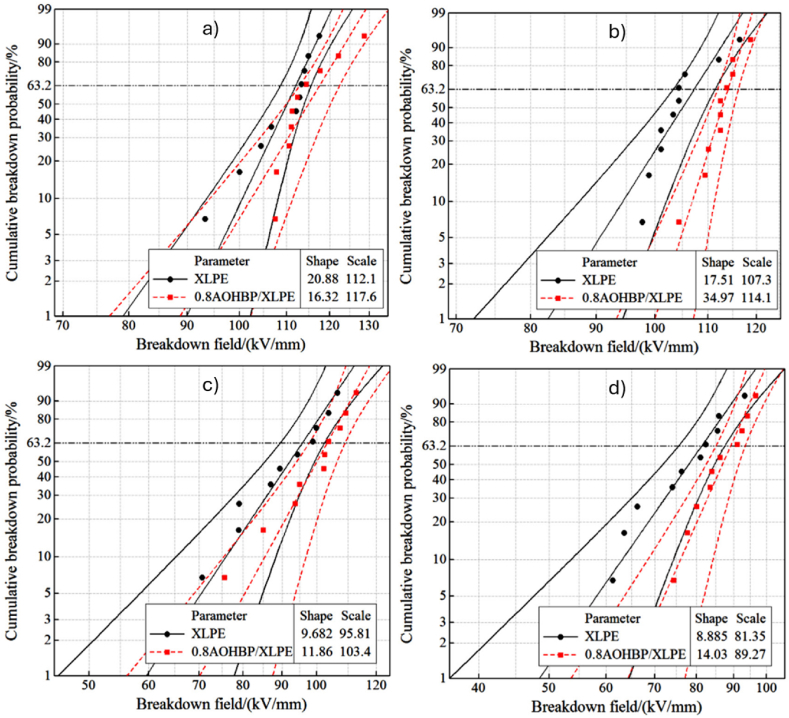
Weibull distribution of AC breakdown strength of XLPE and its composites under different temperatures; a) room temperature, b) 50°C, c) 70°C and d) 90°C [85].

Li et al. [83] compared the effect of three polycyclic aromatic compounds on space charge behaviour of XLPE composites. Unlike the previous studies, the authors directly melt-mixed XLPE with those aromatic compounds but at a minimal loading of 0.5 %. Fig. 11(a1), 11(b1), 11(c1), and 11(d1) indicate the space charge behaviours of neat XLPE, XLPE doped with 4,4′-difluorobenzophenone (PAC-A), 4,4′-dihydroxybenzophenone (PAC-B) and 4,4′-bis (dimethyl amino) benzyl (PAC-C) at 25 °C, respectively. Meanwhile, Fig. 11(a2), 11(b2), 11(c2), and 11(d2) demonstrated the space charge behaviours of neat XLPE, XLPE doped with PAC-A, PAC-B and PAC-C at 80 °C, respectively. At both temperatures, XLPE doped with PAC-C showed lowest space charge accumulation. In the case of Paramane et al. [84], solution blending was employed to mix XLPE with 3-aminobenzoic acid (MABA). The authors expressed that XLPE compounded with 1 % MABA exhibited maximum DC breakdown strength, minor space charge accumulation and the minimum electrical field distortion. Additionally, bare minimum of dielectric constant and dielectric loss were measured. Under the dwell time of 5 min, DC breakdown test recorded time-to-breakdown of 0.5 %, 1 % and 3 % loading of MABA were 631.96 s, 1569.77 s and 613.69 s, respectively. Li et al. [85] disclosed that UV absorber 4-allyloxy-2- hydroxybenzophenone (AOHBP) grafted onto XLPE has an effective effect to the dielectric performance compared to ungrafted one. The results from designed tests showed remarkable enhancements in electrical TIV, inhibition of electrical tree propagation and breakdown strength at different temperatures. Fig. 12 indicates the electrical tree initiation voltage of XLPE and its composites at three different temperatures. Fig. 13(a), (b), and 13(c) elucidate the electrical tree propagation stages of XLPE and its composites under room temperature, 60 °C, and 90 °C, respectively. Fig. 14(a), (b), 14(c), and 14(d) display Weibull distribution of AC breakdown strength of XLPE and its composites at room temperature, 50 °C, 70 °C, and 90 °C, respectively. The effect of AOHBP remains dominant even under long-term AC electric field (6000 h), as indicated by the high AC breakdown strength. The existence of AOHBP in XLPE introduces trapping and excitation effects to scavenge high-energy electrons caused by high local electric field. Thus, the insufficient kinetic energy unable to trigger electron surge for ionisation process.

## 7 Antioxidant Infused XPLE

Oxidation is a degradation mechanism that occurs in polymer materials when highly reactive free radicals interact with them. It can be caused by various factors such as heat, mechanical shear, metallic impurities, and radiation during polymerisation, fabrication or processing. Once initiated, a propagation stage follows, where the reaction between free radical and an oxygen molecule produces peroxyl radical. This peroxyl radical then reacts with an available hydrogen atom within the polymer, forming an unstable hydroperoxide and another free radical. Thus, a continuous chain reaction occurs.

To counteract oxidation, antioxidants (AOs) are added to restrict or delay the propagation steps of oxidation [86]. The performance of antioxidation varies with based on the phenolic compounds present in the AO. Their constituent of monophenols and polyphenols consist of hydroxyl group to retard the free radical oxidation reaction during thermal aging [87,88]. Suraci et al. [89] reported that the radical formation of Irganox 1076 (phenol-based) and Irganox PS802 (thioether-based) produces bigger molecules like hydroperoxide and AO-grafted polymer chains triggering the increase of dielectric losses in the dipolar polarisation frequency region (around 10^5^ Hz). The dielectric spectroscopy response to AO products (dipolar species) is linked to the reduction of oxidation induction time (OIT) as AO conversion intensified under higher dose of aging. In a separate study, the authors [90] added supplementary flame retardant (aluminium hydroxide, ATH) and measured an initial value of accumulated charge density at a significant high value (0.4 C/m^2^) than the uncompounded XLPE (0.05 C/m^2^); attributed to the capability of inserted substances to store charges. Radiation brought forth the size reduction and eradication of AOs and demolition of ATH cluster, which lower the charge store sites concentration leading to the decline of total charge density value. Although ATH accumulation was halted, its chemical properties remained unaffected and able to compensate the trap depth alteration caused by oxidation.

**Fig. 15:**
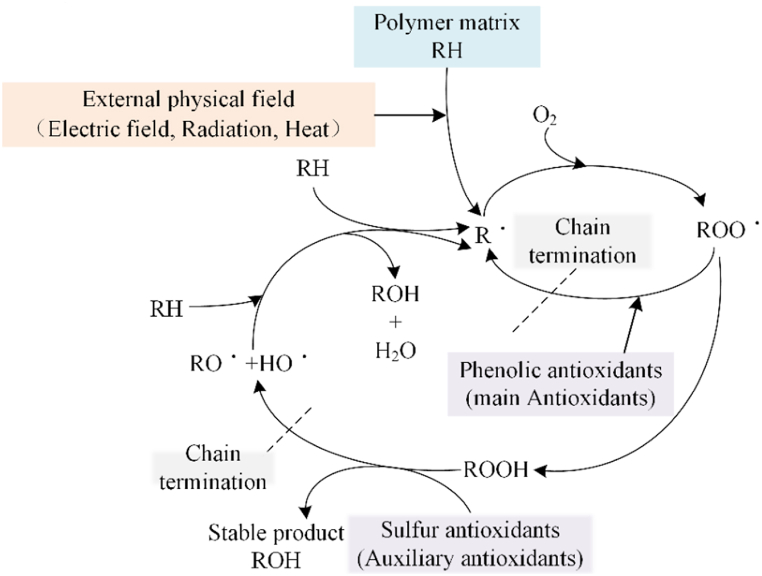
The antioxidant mechanism of E300 [93].

Si et al. [91] indicated that the ortho-substituents of AO, specifically methyl and *tert*-butyl groups, function as electronic groups, augmenting the electron cloud density around the phenolic oxygen and consequently lowering the bond dissociation energy. The molecular structure of AO facilitates the facile separation of the hydrogen atom from the hydroxyl group, serving as a pre-crosslinking inhibitor as AO was incorporated prior to cross-linking process. Pre-crosslinking weakens the integrity of crystalline structure and crystallinity of XLPE, leading to significant increment of injected space charge packet and decrement in the DC breakdown field strength [92]. He et al. [93] injected two type of AOs namely ascorbic acid (E300) and benzoin into aged XLPE cables and discovered that E300 was superior in containing electrical treeing with TIV of 18.53 kV compared 14.42 kV of benzoin. The phenolic group of E300 triggered peroxyl radical (ROO) reaction into hydrogen peroxide (ROOH) and phenol oxygen radical, which responsible to recapture peroxyl radical in mitigating the harmful effects toward the material. In addition, the thioether functional group in E300 acts a radical scavenger to stabilise the decomposition of ROOH into hydroxyl radical attached to an alkyl group (ROH). Fig. 15 displays the chain reaction of E300 in AO mechanism. Meanwhile, Zhang et al. [94] grafted an AO known as N-(4-anilinophenyl) maleimide (MC) onto XLPE to observe its effect on space charge behaviour and DC breakdown strength at manipulated temperatures. Advantageously, the grafting method subdued space charge accumulation, electric field distortion and elevated breakdown strength, particularly at heighten temperatures. The polar groups of MC create immobile and dense deep traps to abstain and scatter charge carriers subsequently minimising charge density and detaining charge migration. Furthermore, the functionality of AO was long-lasting owing to stabilised MC grafted onto XLPE chains. In a different scenario, Liu et al. [95] correlated between DCP and AO loading on the thermal elongation of XLPE. AO hinders the thermo-oxidative aging effect on the macromolecule structure of XLPE to minimise elongation via air heating. The mechanism of antioxidant in preventing elongation depends on hydroxyl number per unit mass or stronger activity of hydroxyl groups to make it performs better. The hydroxyl group present in phenolic antioxidants is responsible in inhibiting the oxidation reaction of free radicals. The greater OIT values of antioxidants signify better antioxidation performance corresponded with lower thermal elongation values. It is noteworthy that higher cross-linking degree does not guarantee to withstand deformation. The elongation values decreased as DCP content increased, while reducing AO content reduction showed slight effects on the upward trend of elongation values. Instead, a collaborative system between the cross-linked network structure and antioxidation performance was necessary to withstand deformation under thermo-oxidation conditions.

The following Equation (3) can be applied to compute the average charge density of nanocomposites for space charge accumulation [96]:

(3) $$q\left(t\right)=\frac{1}{L}\;\int_{0}^{L}|q\left(x,t\right)|dx$$

Where, L is sample thickness, q (x, t) is charge density, x is sample location, and t is polarisation time.

## 8 Polymer combined XLPE

Polymers with complementary properties are often combined with XLPE to enhance its dielectric performance. One such polymer is silicone rubber (SiR), which is a nonlinear conductivity material used to modify the local electrical field and accelerate the dissipation of space charges in polymeric insulation. Typically, a double-layer of insulator structure composed of SiR and other insulating polymers is constructed to redistribute the electrical charges at their contact point, which termed as interface charge accumulation. The major challenge of implementing multi-layered insulation system in power cable application is the cable joint interface, accounting about 80 % of cable failures [97]. The interfacial breakdown occurring between two different insulation materials is not the same as bulk breakdown and surface flashover phenomena. Aside from insulating properties, the contact condition of the interface and filler of the interfacial micro gap also contribute to the breakdown. Kantar et al. [98,99] noted an increment in AC breakdown voltage of the interface as interfacial pressure increases, and the effect of the pressure on the interface is more pronounced when the elastic modulus is lower.

Han et al. [100] doped SiR with SiO_2_ to test reliability of XPLE/SiR composites interface. The incorporation of SiO_2_ leads to a decrease in surface conductivity in the composite, impeding the charge transfer rate on the surface to some extent. This phenomenon increases the likelihood of significant charge accumulation at interface defects. At the flashover test, the 10 % SiO2 doped composite exhibited a lower maximum breakdown voltage but required twice the number of flashovers before the complete failure of interface insulation compared to the unfilled composite. Under applied electric field, positive charges (holes) are readily trapped by SiO_2_ within SiR. The event raised space charge density to a high level and eventually, leads to breakdown when triggered by electrons. SiO_2_ acting as a discharge area were expanded to the entire electrode gap and the varying discharge positions in each event were unlikely to construct a single point breakdown.

**Fig. 16:**
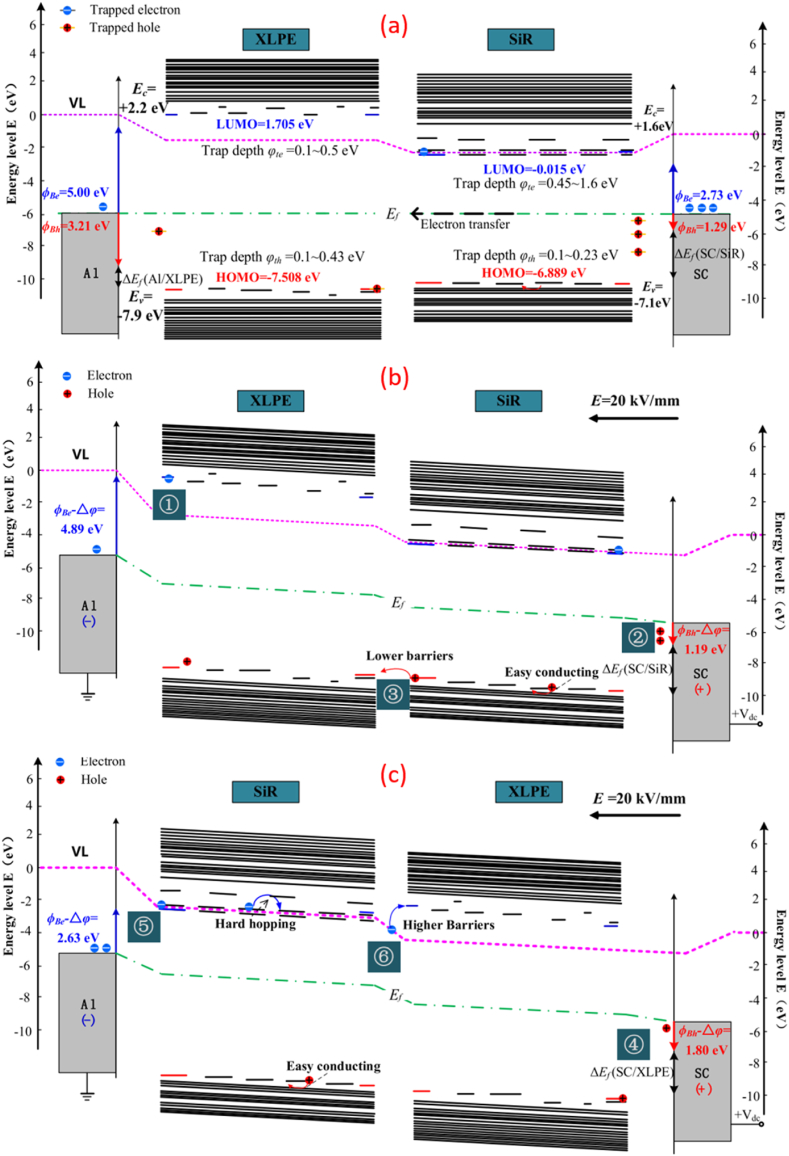
The electronic energy levels of (a) XLPE/SiR without applied voltage (b) + SiR/XLPE- (c) + XLPE/SiR-at 20 kv/mm [101].

Another study by Wang et al. [101] found that the interfacial charges in the +XLPE/SiR-configuration were more pronounced than in the +SiR/XLPE-. Several factors associated with the interfacial charge polarity effect are likely the respective charge traps in XLPE and SiR, interfacial barriers under alternative polarities, variation in the electron and hole injection barriers. It can be observed in Fig. 16(a), under no influence of electric field in XLPE/SiR system, higher Fermi level of SiR instigated electrons migration towards XLPE. In Fig. 16(b), the electron injection barrier in +SiR/XLPE-at 4.89 eV hindered electrons injection into XLPE (1), meanwhile, hole injection barrier at 1.19 eV eased hole injection (2). The existence of shallow hole traps within SiR enables more holes to travel reaching interface (3). Since the disparity of energy levels between localised states on both sides of interface is insignificantly small, impeding effects of interfacial barrier can be neglected. The localized levels precisely situated at the interface are identified as interfacial traps capturing holes' movement on XLPE side, generating positive charge accumulation. In Fig. 16(c), the hole injection barrier in +XLPE/SiR-at 1.8 eV is notably greater (4) than that in +SiR/XLPE-, thus holes are not easily transferred into XLPE from the anode. Meanwhile, the electron injection barrier at 2.63 eV is notably lower than that in +SiR/XLPE-. Additionally, due to the difference in Fermi levels between XLPE and SiR, electrons are more likely to migrate towards the XLPE side. Even so, high interfacial barrier is unfavourable for the electrons’ movement towards the interface (6). The interfacial traps for electrons in the +XLPE/SiR‐ are deeper compared to the interfacial traps for holes in +SiR/XLPE‐ and also, the interfacial barrier is higher in the former configuration. Consequently, +XLPE/SiR‐ exhibits a greater presence of interfacial charges, leading to a comparatively slower charge dissipation during short circuits.

**Fig. 17:**
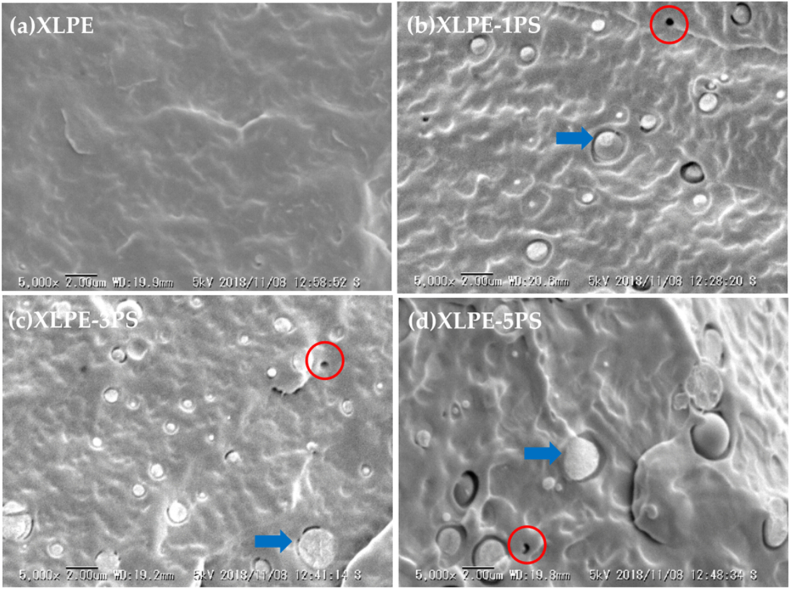
The SEM images of fractured surfaces of (a) neat XLPE, (b) XLPE-1PS, (c) XLPE-3PS, (d) XLPE-5PS [104].

**Fig. 18:**
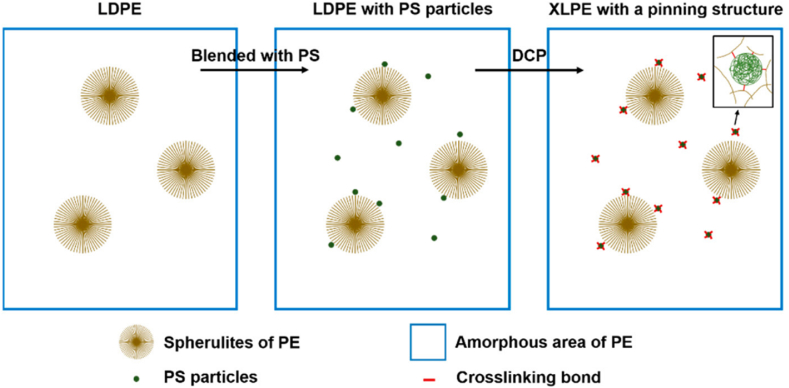
The illustration of XLPE with the addition of polystyrene pinning (PS) [104].

Another promising dielectric material is a synthetic rubber called ethylene propylene diene (EPDM), which is widely used in automotive parts due to its superior resistance against heat, weathering, and aging, as well as its good insulation properties [102]. Wang et al. [103] discovered that bipolar charge dynamics at XLPE/EPDM bilayer interface were ascribed to the charge migration by the electronic energy structure characteristics. Under the positive voltages, the electrons narrowly scatter near the EPDM side, while the holes slowly move towards the interior of the XLPE and form a broad distribution. Progressively, the negative charges (electrons) accumulate and dominate the interfacial charge behaviour. Under the negative voltages, static interfacial positive charges were gathered, which is opposite to the previous set-up. An innovative strategy by Cao et al. [104] by filling XLPE with polystyrene (PS) also bear astonishing results. It was stated that variation loading of PS pinning (1–5 phr) into XLPE significantly increase DC breakdown strength and reduce DC conductivity. The higher loading of PS required higher activation energy (eV) to mobilise charge carriers thereupon result in lower DC conductivity. Fig. 17(a), (b), 17(c), and 17(d) elucidate the morphological structure of neat XLPE, XLPE-1PS, XLPE-3PS, and XLPE-5PS, respectively. Fig. 18 illustrates the establishment of crosslinking network structure through the introduction of PS.

The following equation (4) can be applied to compute interface charge density (σ_i_) at the boundary between two different materials, specifically a polymer (XLPE) and a coating layer [105].

(4) $$\sigma_{i}=\frac{E_{XLPE}\gamma_{coat}-E_{coat}\gamma_{XLPE}}{\gamma_{coat}d_{XLPE}+\gamma_{XLPE}d_{coat}}\;V$$

ℰ_XLPE_ and ℰ_coat_ are the dielectric constants, γ_XLPE_ and γ_coat_ are the conductivities, d_XLPE_ and d_coat_ are the thicknesses of the XLPE and the coating layer, respectively. V is the applied voltage.

## 9 Other additives Infused XLPE

**Fig. 19:**
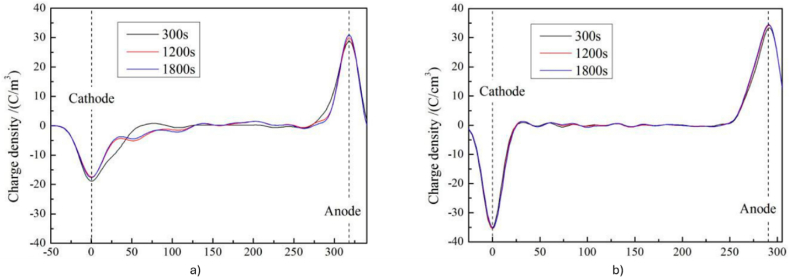
The space charge distribution of a) LLDPE and b) XLPE-g-TMPTMA under applied DC electric field of 40 kV/mm at 25°C [108].

**Fig. 20:**
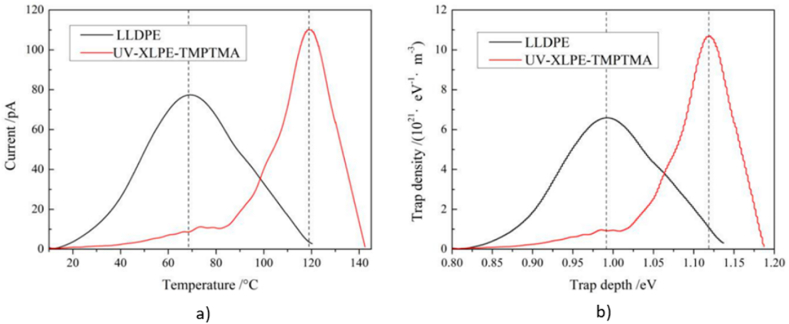
(a) The temperature spectra of thermally stimulated depolarization currents and (b) the distribution of trap level of LLDPE and XLPE-g-TMPTMA [108].

Another effective technique to achieve good dielectric characteristics is by the grafting of polar group molecules into designed polymers. Specific functional groups are introduced into the polymer backbone to modify its properties for better applicability. Prior to XLPE crosslinking, Gao et al. [106] grafted two types of compounds namely chloroacetic acid allyl ester (CAAE) and maleic anhydride (MA) at 0.5 %, 1.0 % and 1.5 % loading. It was revealed that 1.0 % loading produce the highest breakdown strength for both CAAE-g-XLPE and MA-g-XLPE, which are 142.8 kV/mm and 139.6 kV/mm, respectively. Surplus loading ends up with reduction of breakdown strength associated with intensification of electron collision ionisation building up higher electrical field distortions for chance of PD. The grafted polar-group molecules introduced charge traps that resided on a deeper energy level. The maximum temperature at which an effective trapping mechanism can be maintained is influenced by the depth of the trap level. Therefore, when temperature reached upper limit, the charge traps became invalid in suppressing charge carriers due to being thermally excited. The increase concentration of charge carriers in specific regions, creating space charge regions, which altered the local electric field. A similar approach was taken by Zhang et al. [107], who used N, N′-*m*-phenylene dimaleimide (HVA-2) before the XLPE crosslinking process. The crystallinity was diminished as noted by the reduction of breakdown strength. Only at 70 °C, HVA-2 thrive to capture and scatter charge carriers lowering the carrier concentration and mobility consequently increased the breakdown strength but only to a certain degree. Ionisation of impurities, mainly excess of HVA-2 worsen the physical structure of the XLPE thus leading to drop in breakdown strength. Meanwhile, Qiu et al. [108] had supplementary crosslinking agent of trimethylolpropane trimethacrylate (TMPTMA) grafted onto XLPE for the suppression of space charge. Pulsed electro-acoustic (PEA) method signified that XLPE-g-TMPTMA had steady distribution of space charge with a minor accumulation of heterocharges near cathode at the peak value of 1.5 C/m^3^ and the electric field distribution remain stable after 1800 s of complete polarisation process. On the contrary, linear low-density polyethylene (LLDPE) accumulates homocharges near the cathode and the charge density amplifies to the highest value of 4 C/m^3^. Fig. 19(a) and (b) clarify the space charge distributions of LLDPE and XLPE-g-TMPTMA under applied DC electric field of 40 kV/mm at 25 °C, respectively. Thermally stimulated depolarization currents (TSDC) test verified that extra deeper traps were presented in XLPE-g-TMPTMA. Fig. 20(a) and (b) exhibit a new peak in TSDC spectra of XLPE-g-TMPTMA at higher temperature clarifying that extra deeper traps were presented meanwhile the lower temperature peak correlates to the inherent traps due to structural defects of LLDPE. On the other hand, a peculiar strategy by Jarvid et al. [109] reported that the introduction of fullerenes derivative [6,6],-phenyl-C 61 -butyric acid methyl ester (PCBM) into XLPE increased the initial electric field strength of electrical tree by 26 %. The high electron affinity feature owned by fullerenes greatly influenced the charge recombination, where high-energy electrons were scavenged under high electrical field.

## 10 Synergistic effect of voltage stabiliser and nanofiller

Last but not least, the practice of grafting voltage stabiliser onto the surface of nanoparticles is able to enhance the insulation performance under DC electric field. A synergistic effect between these two additives mitigates the migration and precipitation of voltage stabiliser. Jarvid et al. [110] express that the alkylation of voltage stabiliser to some degree can suppress the migration and precipitation but the effect on dielectric properties is trivial. Though, the reliability of this approach still relies on the uniform dispersion of nanoparticles with the polymer matrix [85]. Some authors employed this technique on epoxy, which effectively improve the breakdown strength of the composites. Qin et al. [111] reported that grafted voltage stabiliser namely *m*-aminobenzoic acid (*m*-ABA) onto SiO_2_ within cycloaliphatic epoxy (CE) had its breakdown strength increased by 40.8 % compared to unmodified CE. Meanwhile, Zhang et al. [112] indicated that the breakdown strength of epoxy/*h*-BN improved by 27.4 % when acetophenone was grafted onto *h*-BN. The dielectric properties of material filled with nano-size fillers are greatly influence by the interface effect. The voltage stabiliser grafted nanofillers introduce more interface effects with greater capability to attenuate high-energy electrons through the introduction of deep traps. There have yet study on XLPE reinforcement through this approach.

## 11 Challenges and future Demands

The introduction of various additives, including nanoparticles, voltage stabilisers, antioxidant, and polymers, has been found to enhance the structural system of XLPE and improve its functionality as an insulating material. Nevertheless, there are challenges associated with modifying the original state of the material. The main hurdle lies in securing large-scale fabrication with consistent performance, which is regulated by the compatibility, complexity, and consistency of matrix-filler relationship. In order to fully optimise their interrelation system, certain discrepancies need to be addressed. Firstly, the controllable distribution of nanoparticles within the polymer matrices, a clear understanding of the complexity of the material's structural system, and the feasibility of scaling up the preparation from laboratory-scale to industry-scale. In applications involving high temperatures, multi-layered insulating cables experience different thermal expansions, leading to thermo-mechanical stress. This stress is closely related to changes in the molecular chains, which are influenced by electrical and thermal degradation processes. There is a possibility that compounded additives may leach out at elevated temperatures, reducing their effectiveness or even rendering them ineffective. Conventionally, coupling agents are incorporated to enhance compatibility, but the by-products they leave behind can cause ionisation.

Environmental movement had arisen since the late 19th century due to the global industrial revolution. Over time, electrical insulation materials have evolved from natural rubber to synthetic materials, with the focus shifting away from environmental considerations. In applications that prioritize high durability and efficiency, such as high-voltage cables, only a limited number of materials are suitable. Therefore, it is important to design formulations that address environmental sustainability. In addition to inorganic compounds, bio-based materials can be explored to develop eco-friendly and low-cost alternatives. Plant-based fillers such as nanocellulose and lignin have potential to impart obligatory features like high water resistant, good mechanical strength, enhanced thermal and chemical stability for high performance dielectric material [113,114]. Lignin is a complex phenolic polymer contains antioxidant agent that hinders the oxidation process caused by free radicals. It is often employed as an additive in materials to improve their thermal properties. In actual operation of power transmission, conducting high voltage through the cable lines generated high heat to the insulating layer. As thermal ageing favours oxidation rate, the presence of antioxidant agent in lignin potentially hinders oxidation attack under prolonged heat stress. By applying lignin as a dispersant, metal oxides particles absorb on the lignin's surfaces and alter the overall surface charge density of the suspensions by inducing electrostatic or steric repulsion between particles [115]. Zhou et al. [116] reported that the particle size distribution of titanium oxide modified with alkali lignin was 4 μm compared to titanium oxide alone (25 μm) indicating lesser agglomeration. On the other hand, nanocellulose require grafting of new functional groups to its surface to be applicable in electrical applications. Cyanoethyl cellulose (CEC) exhibiting high dielectric constant, decent heat and acid stability is commonly used as the main matrix reinforced with nanofiller to develop dielectric material. It was reported by Jia et al. [117] that the incorporation of BaTiO_3_ greatly enhances the dielectric permittivity of CEC but signifies minor effect on the dielectric loss. The disparities in chemical properties of both lignin and nanocellulose being polar, while XLPE being non-polar making them to become incompatible. The obvious and conventional approach to promote such contrastive behaviour is either by chemical modification or including coupling agents.

One of the main reasons of extensive efforts in accomplishing high-spec insulator materials is to resist any possible damage and maintain their workability to the utmost degree. Damage inflicted within the cable is practically intractable as it is buried underground and cover wide range. Therefore, embedding self-healing functions in the material is a very valuable prospect for repairing defects [118,119]. Extrinsic approaches, such as implanted microcapsules or vascular-based release of healing agents triggered by crack formation, can activate mechanisms involving activated monomers and active chain ends [120]. Intrinsic approaches rely on the inherent ability of the material to autonomously reconstruct its structure when triggered by external stimuli [121]. Materials possessing intrinsic self-healing properties have reversible bonds capable to break and reform. The chemical bonds involve in the healing mechanism of polymer chains are dynamic non-covalent bonds or dynamic covalent bonds. The viability of both bonds is influenced by kinetic constants and may necessitate external stimuli, including electromagnetic fields, heat, light, and moisture, to enhance chain dynamics and facilitate assembly formation, particularly in the case of covalent bonding. Polymers exhibiting shape memory effects, also known as shape memory polymers (SMPs), possess the ability to self-heal when exposed to specific stimuli such as heat or light [122]. In the case of XLPE that unequipped with inherent self-healing mechanism, extrinsic self-healing mechanism can be implemented. Typically, micro capsule is made of poly (urea-formaldehyde) (PUF) encapsulated healing agent that are made up of catalysts, monomers and resin precursors are embedded in the polymer matrix. Unlike intrinsic self-healing mechanism that require external stimuli to initiate self-healing process, the dispersed micro capsules will break and release the healing agents upon receiving stress, which attract and repair inflicted areas. Another healing agent, dicyclopentadiene (DCPD) was employed by Wang et al. [123] indicated a remarkable self-healing effect on the structural defects of PE caused by mechanical damage (such as scratches) and electrical damage (like electrical treeing), allowing the local high electric field to be homogenized, thereby restoring the insulation strength.

## 12 Summary

Significant efforts have been made to mitigate the risk of dielectric breakdown by tailoring the characteristics of XLPE to enhance its reliability. Various additives have their own intriguing features that contribute to the enhancement of the anticipated products. The interaction between nanoparticles and the matrix phase restricts the mobility of charge carriers, preventing the accumulation of space charge. Silicates, known for their excellent thermal and insulating properties, exhibit exceptional performance at elevated temperatures. However, agglomerated nanoparticles hasten PD to reach ground electrode due to defect in matrix structure inducing charge carrier movement. The polar groups of voltage stabiliser lessen the electron energy, which hinder the ionisation of polymer chain. Antioxidant consisting of hydroxyl group provide decent resistance against thermal aging by inhibiting free-radical oxidation reaction. The grafting method was employed to promote the compatibility of nanoparticle-matrix interaction and prevent the migration of antioxidant and voltage stabiliser compound from the matrix. Special attribute polymer especially silicone rubber can be amalgamated with XLPE to compensate for their flaws. Infusing unconventional additives also able to manipulate the macromolecular chain of the polymer for desired functional properties. Modified XLPE holds a great prospective in high voltage insulation applications. The inclusion of additives imparts advantageous effect in attaining high and consistent performance. Immense research on self-healing polymer is advancing toward dielectric material in effort to preserve the longevity of insulation cable system. The self-healing mechanism, activated by the rupture of microcapsules, is well-suited for high voltage applications where stimuli induce its activation. The inherent properties of biomaterials like lignin and nanocellulose are attractive and can be potentially incorporated into dielectric materials to impart essential characteristics. Yet, challenges remain in scaling up production to higher levels, necessitating a comprehensive understanding of their interrelated systems. The matrix-filler bonding is a complex relationship that dictates a comprehensive insight in securing composite products with consistent performance. An awareness on the type and loading of additives, method of preparation, additives’ characteristics and working mechanism provides valuable information at the stage of material preparation for intended application. These details also represent future investment in acquiring consistent performance of advanced materials systematically.

## Data Availability

Has data associated with your study been deposited into a publicly available repository?

No, Data included in article/supp. material/referenced in article.

## CRediT authorship contribution statement

**Nazrin A:** Writing – original draft, Investigation. **T.M. Kuan:** Validation, Supervision. **Diaa-Eldin A. Mansour:** Writing – review & editing. **Rizwan A. Farade:** Writing – review & editing. **A. Mohd Ariffin:** Conceptualization. **M.S. Abd Rahman:** Methodology. **Noor Izzri Bin Abdul Wahab:** Writing – review & editing.

## Declaration of competing interest

The authors declare that they have no known competing financial interests or personal relationships that could have appeared to influence the work reported in this paper.
